# “Rat race” or “lying flat”? The influence of performance pressure on employees' work behavior

**DOI:** 10.3389/fpsyg.2025.1466463

**Published:** 2025-02-03

**Authors:** Qianyi Liao, Jinsong Zhang, Fangfang Li, Shiyuan Yang, Zhen Li, Longhua Yue, Cunfang Dou

**Affiliations:** School of Public Administration, Sichuan Agricultural University, Ya'an, China

**Keywords:** performance pressure, challenge appraisal, workplace anxiety, proactive work behavior, work withdrawal behavior

## Abstract

**Introduction:**

Performance pressure refers to employees' subjective perception of the necessity to achieve expected goals, accompanied by a sense of urgency and tension. This study explores how employees cope with performance pressure, focusing on two contrasting strategies: the “rat race” (proactive work behavior) or “lying flat” (work withdrawal). Grounded in the transactional theory of stress and affective event theory, this research aims to uncover the mechanisms through which performance pressure influences work behavior.

**Methods:**

A moderated dual-mediation model was developed to examine the dual pathways of challenge appraisal and workplace anxiety as mediators in the relationship between performance pressure and work behavior. Data were collected through a two-stage survey involving 356 employees from various industries. Statistical analyses, including structural equation modeling, were used to test the hypothesized relationships.

**Results:**

The findings reveal that performance pressure has a dual effect: it simultaneously stimulates challenge appraisal, promoting proactive work behavior, and induces workplace anxiety, leading to work withdrawal behavior. Additionally, learning goal orientation moderates these effects. Specifically, it strengthens the positive relationship between performance pressure and challenge appraisal while weakening the link between performance pressure and workplace anxiety.

**Discussion:**

This study highlights the complex and dual nature of performance pressure in influencing employee behavior. By identifying learning goal orientation as a critical moderator, organizations can better understand how to harness the positive aspects of performance pressure while mitigating its negative effects. These insights provide practical guidance for managing performance pressure and minimizing associated risks in the workplace.

## 1 Introduction

To navigate market uncertainties stemming from intense competition, many organizations exert considerable performance pressure on their employees (Tang et al., 2023). These expectations compel employees to complete tasks more quickly, efficiently, and with greater dedication. Concurrently, this intensifies employees' perception of performance pressure. Performance pressure is defined as employees' subjective perception that they must meet established goals (Mitchell et al., 2018). It has become a primary stressor in contemporary workplaces, characterized as the events or event properties (stimuli) encountered by individuals (Zhang et al., 2017). Stressors are ubiquitous in the workplace. Previous studies suggest that performance pressure may result in negative outcomes, including unethical pro-organizational behavior and a reduction in interpersonal citizenship behaviors (Hetrick and Jacobson, 2024). In contrast, other scholars argue that performance pressure can yield positive outcomes, such as fostering job crafting and encouraging innovative behavior (Kumar and Lavanya, 2024). Why do employees exhibit differing responses to performance pressure? What underlying mechanisms account for their varied coping strategies?

This study seeks to address the inconsistencies in the existing literature by proposing a model that examines the relationship between performance pressure and work behavior. Central to our model is an integrated analysis of two distinct types of work behaviors: proactive work behavior and work withdrawal behavior. Proactive work behavior, as defined by Parker and Collins (2008), refers to self-initiated, active, and future-oriented actions aimed at altering the current situation. Work withdrawal behavior refers to the negative behavioral response in which employees distance themselves from the organization (Lehman and Simpson, 1992). Previous studies predominantly relied on a single perspective. For instance, scholars have argued that leadership style and work tasks can promote proactive work behavior (Yu et al., 2023). Simultaneously, research on work withdrawal behavior has predominantly focused on negative antecedents, such as workplace bullying and illegitimate tasks (Fan et al., 2023). This limited perspective overlooks the dual nature of pressure. Given the dual nature of stressors, it is crucial to investigate how stress influences employees' work behaviors. Integrating these two aspects facilitates a more comprehensive understanding of the role of performance pressure.

Recent research has demonstrated that creative leadership significantly impacts employees' proactive work behavior (Zhou et al., 2024). Additionally, studies have highlighted that employees' organizational citizenship behavior affects their work withdrawal behavior (Qian et al., 2020). However, these studies have not thoroughly examined the underlying mechanisms that drive these relationships. Therefore, it is essential to investigate the mediating mechanisms that explain how performance pressure influences employees' work behaviors. This study focuses on uncovering the underlying pathways through which performance pressure drives varying behavioral outcomes among employees. Specifically, it introduces two mediating variables to analyze their roles in shaping these relationships. The transactional theory of stress, introduced by Lazarus and Folkman (1984), underscores the importance of individuals' cognitive appraisal in stress responses. According to this theory, individuals evaluate stressors as either challenges or threats, resulting in distinct emotional and behavioral responses (Lazarus and Folkman, 1984). Challenge appraisal refers to the perception that the current situation fosters personal growth and emphasizes potential gains, growth, or learning (Folkman and Lazarus, 1985; Prem et al., 2017). Lazarus (1999) posits that stress-related emotions may arise in response to stress. The affective event theory offers a robust framework for understanding emotional responses to performance pressure. The theory emphasizes the importance of managing emotional events in daily life to promote the harmonious development of both physical and mental health (Weiss and Beal, 2005). Workplace anxiety, a distinct emotional state (Nerstad et al., 2023), refers to the stress and apprehension individuals experience while performing tasks in a professional setting (Jex, 1998). This study posits that employees who engage in challenge appraisals are more likely to exhibit proactive behaviors. The cognitive appraisal of workplace events plays a crucial role in shaping affective responses (Cheng and McCarthy, 2018). Conversely, when employees perceive events as threats, they may experience increased workplace anxiety, which may ultimately lead to work withdrawal behaviors. This research builds upon and refines previous contributions, including those of Zhou and Qian.

Furthermore, personal dispositions play a crucial role in determining how employees cope with stress. Kundi et al. (2021) suggested that future research should explore how other personal dispositions influence the perception of stressors. Therefore, it is crucial to clarify the role of learning goal orientation in shaping employees' cognitive appraisal and emotional responses. This study examines learning goal orientation as a moderating variable in the relationship between performance pressure, cognitive appraisal, and emotional reactions. Research has indicated that learning goal orientation is a crucial psychological framework for interpreting and coping with challenges (Dweck and Leggett, 1988). It refers to individuals' desire to enhance their work abilities to overcome challenges and complete tasks effectively (Dweck, 1986). Individuals with a high learning goal orientation adopt an adaptive response model characterized by perseverance, a focus on improvement, self-directed problem-solving, and a propensity to embrace challenges (Vandewalle, 1997). When faced with performance pressure, they perceive tasks as challenges and believe they can overcome them, leading to positive cognitive appraisals. Conversely, individuals with a low learning goal orientation are more likely to perceive performance pressure as a threat, which results in workplace anxiety. This research introduces learning goal orientation as a moderating variable to explore factors that influence employees' cognition and emotional responses.

This study presents three key contributions. First, by applying the transactional theory of stress and the affective event theory, we introduce proactive work behavior and work withdrawal behavior as positive and negative coping strategies, respectively. Our findings reveal the “double-edged sword” effect of performance pressure, elucidating why it can sometimes foster proactive work behavior and, at other times, lead to work withdrawal. Second, we elucidate the psychological mechanisms underlying employees' choices between proactive work behavior and work withdrawal behavior in the face of performance pressure. We examine how performance pressure influences work behavior through the incorporation of challenge appraisal and workplace anxiety. This dual-path approach reconciles previous contradictory findings and provides a comprehensive framework for understanding the effects of performance pressure. Third, in response to Mitchell et al. (2019), who highlighted the positive moderating effect of individual traits on performance pressure and called for further exploration of other moderating factors, we examine the role of learning goal orientation. Our research enhances the understanding of the boundary conditions of performance pressure and provides valuable insights for both managers and employees on effectively managing performance pressure.

## 2 Theory and hypotheses

### 2.1 The transactional theory of stress

The transactional theory of stress, proposed by American scholars Lazarus and Folkman (1984), is foundational in the field of stress research. It posits that cognitive appraisal is a key psychological process mediating the relationship between stressors and behavioral responses (Lazarus and Folkman, 1984). Cognitive appraisal is typically divided into two types. From a transactional perspective (Folkman et al., 1986), the first type is primary appraisal, where individuals assign meaning to the situation, evaluate what is at stake, and assess whether the situation poses a potential or actual threat to their wellbeing. In contrast, secondary appraisal refers to the perceived availability of coping resources to address a stressful encounter (Lazarus and Folkman, 1984). During this stage, coping options are evaluated based on available social, personal, economic, and organizational resources, as well as the level of control individuals perceive over the situation (Nerstad et al., 2023). Coping is defined as “cognitive and behavioral efforts to master, reduce, or tolerate the internal or external demands created by the stressful transaction” (Lazarus and Folkman, 1984). Coping behaviors are initiated as a result of primary and secondary appraisals. These processes are interdependent, influencing one another and shaping the nature of each encounter (Lazarus and Folkman, 1984).

Based on the research of Lazarus and Folkman, two primary categories of coping strategies have been identified: problem-focused strategies, which aim to directly address situational demands, and emotion-focused strategies, which aim to manage the emotional responses to those demands (Lazarus and Folkman, 1984). Despite these efforts, however, a lack of consensus persists regarding the optimal classification of coping strategies (Nerstad et al., 2023).

When confronted with performance pressure, employees engage in primary appraisal. They make a challenge appraisal when they perceive the pressure as an opportunity for growth and potential reward. Coping involves addressing either threats or challenges. When individuals appraise stressors as challenges, they typically adopt problem-focused coping strategies, which are associated with positive behaviors and outcomes. Individuals who perceive stressors as challenges are more likely to adopt proactive coping strategies. Such individuals exert more effort and perform better than those who perceive stressors as threats (Lu et al., 2023). Consequently, we hypothesize that challenge appraisals are positively associated with proactive work behavior.

Individual traits play a significant role in shaping how individuals appraise stress (Lazarus and Folkman, 1984). This perspective enables the exploration of moderating variables that influence stress appraisal. One such trait, learning goal orientation, significantly influences how employees evaluate and respond to stressors (Lu et al., 2023). In this context, varying levels of learning goal orientation may lead to differing appraisals of performance pressure. This study explores how individuals with differing levels of learning goal orientation perceive performance pressure and how these perceptions influence work outcomes. Overall, this theoretical framework elucidates the beneficial influences of performance pressure on employee behavior and emphasizes the role of learning goal orientation in understanding these influences.

### 2.2 The Affective Events Theory

The Affective Events Theory (AET), developed by Weiss and Cropanzano (1996), emphasizes that an individual's affective responses to workplace events influence their moment-to-moment cognition and behavior (Ashton-James and Ashkanasy, 2005; Weiss and Cropanzano, 1996). Consequently, the workplace functioning of organizational members is more reliably predicted by their affective responses to workplace conditions (Ashton-James and Ashkanasy, 2005). Affect not only influences the way information is processed but also shapes the content of judgments and evaluations (Ashton-James and Ashkanasy, 2005). While the transactional theory of stress also considers emotional reactions, its primary focus is on the generation of stress and coping strategies (Folkman et al., 1986). The AET is a crucial extension of the existing theoretical framework designed to explain and predict how individuals experience, express, and regulate their emotions in specific situations (Weiss and Beal, 2005). Work events can be classified into two categories: troublesome or adverse events that hinder the achievement of work goals and elicit negative emotions, and exciting events that facilitate work goals and generate positive emotions (Weiss and Cropanzano, 1996). Additionally, cognitive appraisals of events precede emotional reactions (Weiss and Cropanzano, 1996). Thus, performance pressure, when perceived as a threat to achieving personal goals, is considered a negative work event that may trigger workplace anxiety. Emotional reactions in the workplace play a significant role in influencing employee behavior (Ashton-James and Ashkanasy, 2005). Emotionally driven behavior refers to responses that are directly shaped by intense emotions. Based on this perspective, we hypothesize that workplace anxiety, driven by emotional responses, may lead to work withdrawal behaviors.

The AET also posits that individual traits influence emotional reactions (Weiss and Beal, 2005). The theory identifies two key personality traits: positive affectivity and negative affectivity. Individuals with high positive affectivity are generally enthusiastic and often experience positive emotions, focusing on favorable work events and reacting more positively. Conversely, individuals with high negative affectivity are more prone to experiencing depression and tend to focus on negative aspects, leading to more negative emotional reactions (Weiss and Beal, 2005). Building on this idea, we propose that learning goal orientation, as an optimistic individual trait, can mitigate the negative impact of performance pressure on workplace anxiety. AET elucidates the emotional mechanisms underlying workplace behavior, helping to clarify how performance pressure negatively impacts employee behavior. Furthermore, it emphasizes the significance of learning goal orientation as a critical individual trait in these processes. This study extends the applicability of AET by incorporating learning goal orientation.

**Figure 1:**
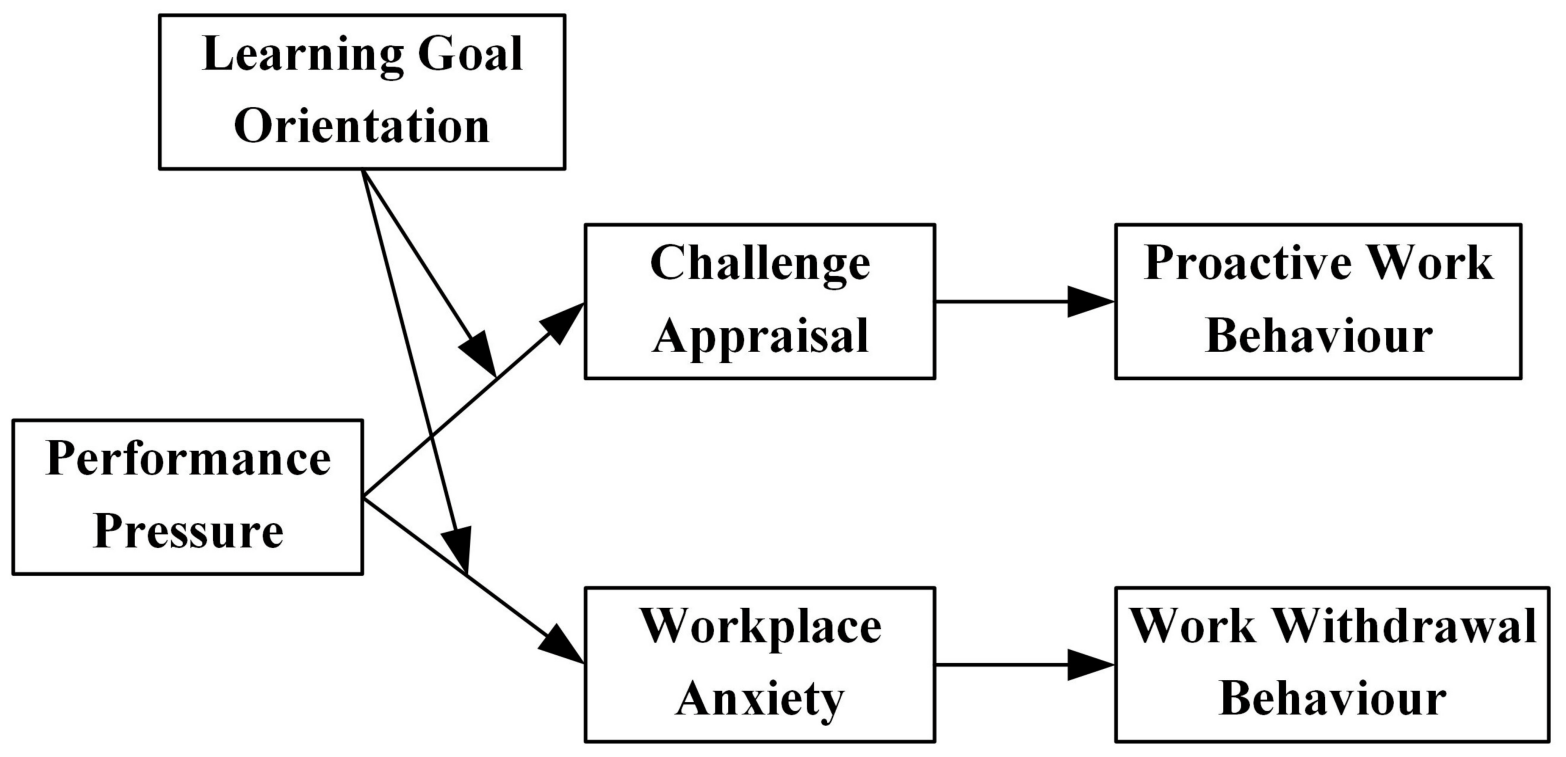
Theoretical model.

Therefore, this study develops a moderated mediation framework grounded in the transactional theory of stress and the AET, as depicted in Figure 1.

### 2.3 Performance pressure, challenge appraisal, and proactive work behaviors

Performance pressure refers to employees' perception that they must meet specific goals, creating a sense of urgency and tension (Mitchell et al., 2018). Lazarus and Folkman (1984) identified two types of cognitive appraisals of stressful situations: challenge appraisal and threat appraisal. Challenge appraisal refers to individuals' belief that they can manage and even thrive under stressors (Lazarus, 1991). In organizational settings, employees often perceive performance pressure as a challenge (Mitchell et al., 2019). This perception suggests that individuals view performance pressure as something they can overcome, which positively impacts both their work performance and personal growth (Lepine et al., 2005). Consequently, employees are able to enhance their work efficiency and complete tasks more promptly. Furthermore, performance pressure includes leaders' high expectations and demands for employee performance. Research indicates that these high-performance demands can catalyze employee development (Wahab and Tatoglu, 2020). Ko and Choi (2019) further argue that when employees perceive this pressure as an opportunity for career advancement and personal growth, high-performance expectations can substantially enhance employee performance. Therefore, performance pressure can be a beneficial stressor, promoting employee growth and enhancing performance.

Additionally, challenge appraisal refers to individuals perceiving their environment as conducive to self-development, emphasizing potential gains, growth, and learning (Folkman and Lazarus, 1985; Prem et al., 2017). This mindset typically arises in stressful situations that present intrinsic benefits or opportunities for growth (Lazarus and Folkman, 1987). When confronted with demanding circumstances, individuals who believe they can manage them effectively tend to view the situation as a challenge (Lazarus and Folkman, 1984). Successfully managing performance pressure often results in feelings of accomplishment and personal development (Lepine et al., 2005), fostering a positive interpretation of performance pressure. Based on these insights, we propose the following hypothesis:

H1: Performance pressure positively predicts challenge appraisal.

An individual's cognitive appraisal plays a significant role in shaping their subsequent behavioral responses (Lazarus and Folkman, 1984). When individuals perceive stressors as challenges, they are more likely to employ problem-focused coping strategies (Lazarus and Folkman, 1984). Research indicates that such challenge appraisals are associated with positive outcomes, including fostering individual responsibility orientation and facilitating job crafting (Zhang and Parker, 2022). Proactive work behavior, characterized by spontaneity, foresight, and transformation (Parker and Collins, 2008), is crucial for achieving organizational goals. This study posits that proactive work behavior reflects individuals' approach to problem-solving in response to stressors. Challenge appraisal, wherein individuals view stressors as manageable opportunities, is expected to positively influence proactive work behavior. Previous research has consistently shown that challenge appraisals motivate individuals to actively address challenges, with empirical evidence supporting a positive correlation (Carenzo et al., 2020). When individuals adopt a challenge appraisal at work, they leverage existing resources more confidently (Lazarus and Folkman, 1984), focusing intensely on their goals and taking practical steps to achieve them. Mitchell et al. (2019) suggested that perceiving performance pressure as a challenge activates individuals' internal resources, enhancing their engagement. Consequently, it can be inferred that when individuals confront performance pressure with a challenging mindset, they are more likely to proactively refine their work approaches, tackle challenges, and exhibit greater proactive behaviors. Based on these insights, we propose the following hypothesis:

H2: Challenge appraisal positively predicts proactive work behaviors.

Cognitive appraisal serves as a psychological mediator between stressors and their subsequent outcomes for individuals. Employees who experience performance pressure evaluate it from challenge or hindrance perspectives. This appraisal depends on their judgment of whether they have the necessary coping strategies to effectively manage the pressure. When individuals successfully navigate performance pressure, they often experience a sense of achievement and personal growth (Lepine et al., 2005), which reinforces a challenge appraisal. Furthermore, individuals' cognitive appraisals of performance pressure influence their proactive work behaviors. Challenge appraisal encourages individuals to invest greater effort in problem-solving at work. Coping, fundamentally, involves how individuals interact with and manage the demands imposed upon them by their environment (Lazarus and Folkman, 1987). When confronted with demanding situations, individuals who believe in their ability to manage them are likely to appraise them as challenges. Empirical studies provide evidence that challenge appraisal serves as a mediating factor in various contexts. For example, Prem et al. (2017) demonstrated that challenge appraisal partially mediates the relationship between time pressure and work engagement. Similarly, Lavoie et al. (2021) emphasized the mediating role of challenge appraisal in the relationship between stressors and emotional responses. This highlights the importance of challenge appraisal as a critical mechanism linking stressors to their outcomes. Building on the logical connections between hypotheses H1 and H2, we propose the following hypothesis:

H3: Challenge appraisal mediates the relationship between performance pressure and proactive work behavior.

### 2.4 Performance pressure, workplace anxiety, and work withdrawal behaviors

Workplace anxiety refers to the tension and stress employees experience while completing tasks and striving to meet performance expectations (McCarthy et al., 2016). According to the AET, performance pressure may be perceived as a threat, representing a negative event in the workplace (Lemer and Keltner, 2001). High-performance pressure entails significant risks and demands, leading employees to perceive that their goals or interests are threatened. These threats often evoke anxiety. Performance pressure may lead individuals to doubt their ability to meet expectations, resulting in negative emotions such as anxiety (Nieuwenhuys and Oudejans, 2017). Empirical research by Cui and Li (2021) demonstrates a strong relationship between workplace anxiety and work-related stress. Employees under performance pressure may fear falling short of expectations, potentially leading to negative outcomes such as criticism or reprimands. This anxiety drains their cognitive and emotional resources, impairing effective problem-solving. Furthermore, performance pressure can create a perceived lack of control, overwhelming employees and undermining their confidence in completing tasks. These feelings exacerbate anxiety by further depleting resources.

In addition, during secondary appraisal, employees often perceive performance pressure as an obstructive stressor (Cheng and McCarthy, 2018). This perception stems from two key aspects: First, the organization's emphasis on high performance entails stringent task standards and demands. These high expectations carry significant risks and threats, requiring substantial time and effort. Confronted with the threat of resource depletion, employees may prioritize conserving their resources, resulting in negative emotions such as anxiety and tension. Second, employees seek recognition and acceptance within the organization's social environment, and poor performance is often met with disapproval or rejection (Mitchell et al., 2018). Performance pressure emphasizes past efforts and perceived insufficient performance, leading employees to feel that overcoming these challenges is difficult (Mitchell et al., 2019). As a result, employees are more likely to experience workplace anxiety. Based on these findings, we propose the following hypothesis:

H4: Performance pressure positively predicts workplace anxiety.

The AET posits that workplace events influence employees' emotional responses and behaviors (Weiss and Cropanzano, 1996). Anxiety, a fundamental biological response, is an integral component of the human defense mechanism. For most individuals, anxiety is a detrimental emotional state that diverts attention, impairing performance. When confronted with perceived threats, anxiety mobilizes an individual's resources to protect them, facilitating escape or avoidance. As a result, employees experiencing anxiety are more likely to engage in work withdrawal behaviors (Yin et al., 2023). Previous research has predominantly focused on the effects of work-related anxiety on employee behavior and performance. Research has shown that workplace anxiety decreases work engagement, lowers work performance, and impedes innovation (Gan et al., 2023). Raza et al. (2021) argued that workplace anxiety can contribute to employee turnover, a view supported by Yin et al. (2023), who also associate it with work withdrawal behavior. Moreover, anxiety depletes employees' work resources and triggers withdrawal behaviors when they encounter anxiety-inducing situations. Based on these findings, the present study proposes the following hypothesis:

H5: Workplace anxiety positively predicts work withdrawal behavior.

As performance pressure increases, employees may experience heightened anxiety, which can impact their cognition and behavior (Nieuwenhuys and Oudejans, 2017). According to the AET, negative work behaviors frequently follow negative emotional responses. Stress and frustration at work are primary triggers for these negative emotional responses. Anxiety, a defensive emotional mechanism, is activated by perceived threats. When employees perceive threats to their goals or interests, they frequently respond with negative emotions. These negative emotional responses can result in passive behaviors and retaliatory actions, including task evasion and organizational avoidance via work withdrawal behaviors.

Given the interconnections between performance pressure, workplace anxiety, and work withdrawal behavior, performance pressure can be conceptualized as a hindering stressor. Performance pressure depletes employees' energy and attention, thereby contributing to workplace anxiety. To alleviate workplace anxiety, employees may engage in work withdrawal behaviors as a means of recovering lost resources and mitigating its negative impacts. Based on this, this study proposes the following hypothesis:

H6: Workplace anxiety mediates the relationship between performance pressure and work withdrawal behavior.

### 2.5 The moderating role of learning goal orientation in the relationship between performance pressure and challenge appraisal

Learning goal orientation refers to an individual's motivation to enhance their skills in order to effectively solve problems and complete tasks. This orientation reflects an individual's drive to improve their abilities by acquiring new skills and mastering new challenges (Dweck, 1986). Learning goal orientation is generally considered a personal disposition (Button et al., 1996), and is associated with the belief that significant effort is productive. This is because such effort helps activate and enhance one's abilities, ultimately leading to optimal performance (Vandewalle et al., 2019). Individuals with a learning goal orientation tend to exhibit adaptive responses when they are confident in their abilities (Vandewalle et al., 2019). The transactional theory of stress emphasizes the role of individual traits in shaping the cognitive appraisal of stressors (Lazarus and Folkman, 1984). Therefore, we propose that learning goal orientation moderates the relationship between performance pressure and challenge appraisal. Employees with high learning goal orientation are more likely to actively engage with challenging work and seek strategies to manage tasks (Harish et al., 1994). Performance pressure often introduces complexity and challenge, which individuals with high learning goal orientation are more likely to find motivating. These individuals are not overly concerned by mistakes and place a high value on personal growth and control over their work (Harish et al., 1994; Vandewalle, 1997). Consequently, such employees are likely to perceive performance pressure as an opportunity for personal development.

Furthermore, individuals with a positive outlook are more likely to engage in challenge appraisals. Employees with high learning goal orientation view performance pressure as an opportunity to enhance their capabilities (Lu et al., 2023), responding with positive emotions and increased motivation (Vandewalle et al., 2019). They tend to experience a heightened sense of personal control, which increases their confidence in overcoming challenges and motivates them to adopt challenge appraisals. This trait is aligned with the career development potential of performance pressure, fostering increased vitality and enthusiasm for learning. In contrast, employees with low learning goal orientation are less able to overlook the negative aspects of performance pressure, such as high-performance demands from leaders. As a result, they are more likely to perceive performance pressure as a threat. Based on the above discussion, we hypothesize that learning goal orientation moderates the relationship between performance pressure and challenge appraisals. Therefore, we propose the following hypothesis:

H7: Learning goal orientation positively moderates the relationship between performance pressure and challenge appraisal.

Integrating hypotheses H3 and H7, this study proposes a moderated mediation effect. This study posits that learning goal orientation not only moderates the relationship between performance pressure and challenge appraisal but also moderates the indirect effect of challenge appraisal on the relationship between performance pressure and proactive work behavior. A higher level of learning goal orientation enhances the effect of performance pressure on challenge appraisal, thereby enabling employees to proactively respond to challenges and fostering proactive work behavior (Button et al., 1996).

H8: Learning goal orientation strengthens the mediating influence of challenge appraisal between performance pressure and proactive work behavior.

### 2.6 The moderating role of learning goal orientation in the relationship between performance pressure and workplace anxiety

The AET posits that individual traits influence employees' emotional responses (Weiss and Cropanzano, 1996). Employees with high positive affective traits tend to exhibit positive emotional responses, which can mitigate the negative perceptions associated with adverse events. Conversely, individuals with high negative affective traits are more likely to exhibit negative emotional responses to such events (Weiss and Cropanzano, 1996). These individual traits significantly influence emotional states and other work-related attitudes (Weiss and Cropanzano, 1996). Learning goal orientation, which is characterized by a focus on self-improvement and development, is considered a positive psychological trait. Employees with a high learning goal orientation are intrinsically motivated to learn and grow. They invest effort and persist in completing complex tasks without relying on external rewards (Dweck and Leggett, 1988). They prioritize the process of the task over its outcome, as engaging in the work offers opportunities for skill enhancement and knowledge expansion. In a learning-oriented environment, success is defined as progress, and failure is viewed as an integral part of the learning process. Such an environment fosters continuous learning and skill development. Employees with a high learning goal orientation view performance pressure as an opportunity to enhance their capabilities (Lu et al., 2023), responding with positive emotions and increased motivation (Vandewalle et al., 2019). Precise goal-setting and feedback can help employees better understand their performance and identify areas for improvement, thereby reducing uncertainty regarding performance pressure and alleviating workplace anxiety. In contrast, employees with a low learning goal orientation are more likely to doubt their ability to handle pressure. They perceive performance pressure as a setback or threat. They consider performance pressure detrimental, prioritizing error and risk avoidance. The learning and development opportunities associated with performance pressure do not align with their needs, resulting in increased workplace anxiety.

When confronted with performance pressure, employees with a high learning goal orientation seek to enhance their abilities to manage external demands, as they tend to approach situations with less fear and anxiety and possess more constructive beliefs about the value and significance of effort following setbacks (Vandewalle et al., 2019). By setting specific, achievable learning goals, they can more effectively utilize their resources to cope with challenges, thereby reducing anxiety. Based on these viewpoints, this study proposes the following hypothesis:

H9: Learning goal orientation negatively moderates the relationship between performance pressure and workplace anxiety.

By integrating hypotheses H6 and H9, a moderated mediation effect is proposed. This study posits that learning goal orientation not only moderates the relationship between performance pressure and workplace anxiety, but also moderates the indirect effect of workplace anxiety on the relationship between performance pressure and work withdrawal behavior. A higher level of learning goal orientation will mitigate the depletion of cognitive resources caused by workplace anxiety due to performance pressure, a stressor, and subsequently reduce anxiety levels, thereby diminishing work withdrawal behavior. Based on these viewpoints, this study proposes the following hypothesis:

H10: Learning goal orientation weakens the mediating influence of learning goal orientation between performance pressure and work withdrawal behavior.

## 3 Materials and methods

### 3.1 Sample and data collection

We utilized Credamo, a professional data collection platform, to administer an online survey targeting employees from state-owned enterprises, public institutions, and private enterprises in China. The state-owned enterprises primarily consisted of large-scale organizations operating in industries such as energy and finance. Public institutions included organizations from the education and healthcare sectors, while private enterprises represented industries such as technology, manufacturing, and retail. Most private enterprises were small to medium-sized enterprises (SMEs), whereas the state-owned enterprises and public institutions were predominantly large organizations with over 5,000 employees. The rationale for this selection is as follows: (1) Workplace anxiety is prevalent across all types of enterprises; (2) Regardless of enterprise type, employees are expected to achieve high performance, thereby creating value and enhancing organizational competitiveness in the market; (3) The study focused on formal employees as research participants. Regular employees typically exhibit a strong sense of responsibility and commitment to the organization, and their work withdrawal behaviors may have a more significant impact on organizational outcomes. Moreover, the work withdrawal behaviors of regular employees are often more observable and quantifiable, which enhances the operational feasibility and accuracy of the study.

In the questionnaire, we initially informed respondents that the survey was anonymous. We then provided participants with an overview of the study's purpose and methodology (i.e., the study was conducted in two waves), ensuring strict adherence to relevant laws regarding the confidentiality of their personal information. In accordance with the recommendations of Aguinis et al. (2020), we implemented the following measures to enhance the effectiveness of online data collection. First, we established clear criteria for selecting suitable participants (i.e., employed individuals with high credit scores recorded on the data platform). Second, we performed an attention check. We employed reverse and logical test questions to ensure participants engaged with all items in the survey. Participants received monetary compensation, ranging from 5 to 20 yuan, as a reward for their participation in the study. Subsequently, we excluded questionnaires deemed invalid based on specific criteria. These criteria included missing data, unusually short completion times, duplicated responses, and incorrect answers to logical test questions. Finally, we selected employees from China, encompassing state-owned enterprises, public institutions, and private enterprises, as research participants, given the prevalence of performance pressure in these sectors.

To mitigate common method bias, we conducted two waves of data collection with a 3-week interval, consistent with the multi-round design methodology used in previous studies (Kumar and Lavanya, 2024). Podsakoff et al. (2012) recommended this time separation approach, which reduces the likelihood of respondents using previous answers to fill memory gaps by allowing previously recalled information to dissipate from short-term memory. The study consisted of two phases of data collection. During the data collection process, we administered two different questionnaires to the same group of respondents, collected the last four digits of participants' mobile phone numbers for matching purposes, and informed them that they would receive a reminder for the second round of surveys. We recruited a total of 450 participants. The data were collected for ~3 weeks, from February 15 to March 11, 2024. In the first phase (T1), conducted on February 15, 2024, an online survey was administered to 450 participants, collecting data on their basic information, performance pressure, and learning goal orientation. Following this, we conducted a preliminary screening of the collected responses based on the aforementioned criteria and excluded 49 questionnaires. Three weeks later, on March 11, 2024, the second phase (T2) was initiated. During this phase, 401 participants completed a follow-up survey designed to measure challenge appraisal, workplace anxiety, proactive work behavior, and work withdrawal behavior. However, 30 participants failed to respond to the survey. As a result, the dataset used for analysis included data from 371 participants. After thoroughly examining the second-stage questionnaires, we cleaned the data based on the previously established criteria, resulting in 356 valid questionnaires for analysis. Data from these 356 participants were considered reliable and were subsequently included in the analysis. We explicitly stated that the dataset has not been used in previous analyses.

The gender distribution of the survey subjects was nearly equal, with females representing 51.1% and males representing 48.9%; The age distribution was as follows: 12.1% were under 25 years old, 39.3% were between 26 and 35 years old, 26.4% were between 36 and 45 years old, 14.0% were between 46 and 55 years old, and 8.1% were over 56 years old; Regarding educational background, 44.9% held a bachelor's degree, 22.5% held a junior college degree, 9.6% held a master's degree or higher, 14.3% had completed high school or technical secondary school, and 8.7% had a junior high school education or lower; Regarding enterprise types, private enterprises represented 43.3%, state-owned enterprises represented 29.8%, public institutions represented 26.9%; Regarding job level, grassroots managers represented 37.1%, middle-level managers represented 32.9%, general employees represented 22.8%, and senior managers represented 7.3%; Regarding years of work experience, 29.8% had between 6 and 10 years, 27.5% had between 1 and 5 years, 21.6% had more than 15 years, and 18.0% had between 11 and 15 years.

### 3.2 Measures

This study employed well-established scales that have been validated by numerous scholars both domestically and internationally to measure all key variables. For all English-language scales, this study employed a translation-back translation procedure (Berry, 2002). This procedure aims to preserve the original intent of the scale items while adapting them to the linguistic and cultural norms of the Chinese context. All scales employed a 5-point Likert scale, with 1 indicating “very inconsistent” and 5 indicating “very consistent.” The questionnaire comprised a total of 40 items. The following scales were selected:

#### 3.2.1 Performance pressure

This study utilized the scale developed by Ivancevich and Matteson (1984). The scale categorizes work pressure into several dimensions, including work requirements, interpersonal relationships, and work rewards, comprising 7 items. Representative items include, “I feel that my knowledge structure cannot meet performance requirements” and “To achieve performance goals, I must reduce or avoid mistakes and deviations.”

#### 3.2.2 Challenge appraisal

This study employed the 3 item challenge appraisal scale developed by Lepine et al. (2005). Representative items include, “Overall, I feel my work promotes my achievements.”

#### 3.2.3 Workplace anxiety

This study utilized the 8-item scale developed by McCarthy et al. (2016). Representative items include, “I feel overwhelmed by poor work performance” and “I often feel anxious because I cannot complete work tasks within the specified time.”

#### 3.2.4 Proactive work behavior

This study employed the scale developed by Frese et al. (1997), which consists of 5 items. Representative items include, “I will actively deal with work problems” and “I will try to seize opportunities to achieve my work goals.”

#### 3.2.5 Work withdrawal behavior

This study utilized the work withdrawal behavior scale developed by Lehman and Simpson (1992). The items were integrated into a 6 item unidimensional scale, reflecting the subjects' interpretations and perceptions. For instance, “nap during work hours” and “consider leaving the job during work” were combined into, “I sometimes nap or consider leaving the job during work.”

#### 3.2.6 Learning goal orientation

This study employed the scale developed by Vandewalle (1997), comprising 5 items. Representative items include, “I am willing to choose challenging tasks with more learning opportunities” and “I often seek opportunities to learn new skills and knowledge.”

#### 3.2.7 Control variables

To mitigate bias in the research results arising from individual factors, this study considers employees' position, education, gender, and age, as these may influence their responses to work stressors (Porath and Pearson, 2012). Therefore, this study gathered basic demographic information from respondents, including gender, age, education, company type, position level, and years of work experience, using these variables as control factors. The study controlled for these variables to enhance the explanatory power of the model.

In the questionnaire design, gender was measured using the following scale: 1 = “male” and 2 = “female.” Age was measured using a five-point scale: 1 = “under 25 years old,” 2 = “26–35 years old,” 3 = “36–45 years old,” 4 = “46–55 years old,” and 5 = “over 56 years old.” Education was measured using a five-point scale: 1 = “junior high school and below,” 2 = “high school/technical secondary school,” 3 = “college,” 4 = “undergraduate,” and 5 = “graduate student and above.” Enterprise type was measured using a 5-point scale: 1 = “private enterprise,” 2 = “state-owned enterprise,” 3 = “foreign-funded enterprise,” 4 = “government or public institution,” and 5 = “other.” Position level was measured using a 4-point scale: 1 = “ordinary employee,” 2 = “grassroots manager,” 3 = “middle manager,” and 4 = “senior manager.” Work experience was measured using a four-point scale: 1 = “5 years or less,” 2 = “6–10 years,” 3 = “11–15 years,” and 4 = “more than 16 years.”

### 3.3 Analysis strategy

Data analysis for this study was conducted using SPSS 27.0 and Mplus 8.3 software. First, a data quality test was conducted to ensure that the questionnaire data met the required analytical standards. Subsequently, descriptive statistics and correlation analyses were performed to preliminarily examine the relationships among the key variables, establishing a foundation for the subsequent regression analysis. Finally, all hypotheses were tested using hierarchical regression analysis and the PROCESS macro in SPSS.

## 4 Empirical results

### 4.1 Descriptive statistics of variables

An overall analysis of the research variables was conducted by examining the mean (M), standard deviation (SD), variance, maximum value, minimum value, skewness, and kurtosis for each variable in the sample data. Descriptive statistics for the six variables were obtained using SPSS 27.0 software. As shown in Table 1, the maximum and minimum values of each variable range from 1 to 5. The means are concentrated between 3.313 and 3.397, all within a reasonable range. The standard deviation and variance for the variables are generally <1, indicating good sample stability. According to Kline (2023), the absolute skewness should be <2, and the absolute kurtosis should be <7. The absolute skewness for each variable in this study is <2, and the absolute kurtosis is <7, satisfying the criteria for skewness and kurtosis. These results meet the conditions for a normal distribution and allow for further analysis in the next step.

**Table 1:** Descriptive statistics of variables.

	**M**	**SD**	**Skewness**	**Kurtosis**	**Minimum**	**Maximum**
PP	3.292	0.918	0.079	−1.011	1.430	5.000
WA	3.320	0.906	0.038	−0.912	1.380	5.000
CA	3.374	0.977	−0.068	−0.963	1.330	5.000
WWB	3.325	0.920	0.019	−0.979	1.330	5.000
PWB	3.270	0.973	0.090	−1.099	1.200	5.000
LGO	3.378	1.012	−0.110	−1.155	1.200	5.000

PP, performance pressure; CA, challenge appraisal; WA, workplace anxiety; PWB, proactive work behavior; WWB, work withdrawal behavior; LGO, learning goal orientation.

### 4.2 Confirmatory factor analysis

To assess discriminant validity, we performed confirmatory factor analysis (CFA) using Mplus 8.3 software on six constructs: performance pressure, challenge appraisal, workplace anxiety, work withdrawal behavior, proactive work behavior, and learning goal orientation. We used χ^2^/df, RMSEA, SRMR, CFI, and TLI as evaluation metrics. Our baseline model was a six-factor model, which we compared against several alternative models. The results, presented in Table 2, indicate that the six-factor model outperforms all alternative models in terms of data fit, fully satisfying widely accepted academic criteria (χ^2^/df = 1.159 < 3, RMSEA = 0.021 < 0.05, SRMR = 0.033 < 0.05, CFI = 0.988 > 0.9, TLI = 0.986 > 0.9). This supports the high discriminant validity of the six constructs within the data.

**Table 2:** Confirmatory factor analysis.

**Model**	**Factor**	**χ^2^**	**df**	**χ^2^/df**	**RMSEA**	**CFI**	**TLI**	**SRMR**
	Six factors + potential methodological factors	584.036	511	1.143	0.020	0.989	0.988	0.033
Six-factor model	PP, CA, WA, LGO, PWB, WWB	593.465	512	1.159	0.021	0.988	0.986	0.033
Five-factor model	PP, CA+WA, LGO, PWB, WWB	931.973	517	1.803	0.047	0.937	0.931	0.062
Four-factor model	PP, CA+WA, LGO, PWB+WWB	1453.084	521	2.789	0.071	0.857	0.846	0.078
Three-factor model	PP, CA+WA+LGO, PWB+WWB	2380.054	524	4.542	0.100	0.716	0.696	0.106
Two-factor model	PP+CA+WA+LGO, PWB+WWB	3363.791	526	6.395	0.123	0.566	0.537	0.122
Single-factor model	PP+CA+WA+ LGO+PWB+WWB	3908.105	527	7.416	0.134	0.483	0.449	0.127

PP, performance pressure; CA, challenge appraisal; WA, workplace anxiety; LGO, learning goal orientation; PWB, proactive work behavior; WWB, work withdrawal behavior.

To further assess the convergent validity of the scale, we used Mplus 8.3 software to perform CFA on a dataset of 356 samples. We selected average variance extracted (AVE) and composite reliability (CR) as indicators of internal fit validity for our measurement model. A higher AVE value reflects stronger correlations among indicators of the same construct, while a higher CR value suggests greater consistency in the measurement indicators' ability to explain the variable. Typically, the CR value should exceed 0.6 (Bacon et al., 2016). Regarding the AVE value, Fornell and Larcker (1981) suggested that an AVE value >0.5 indicates a good result, while values between 0.36 and 0.5 are considered acceptable. Based on these criteria, we calculated the AVE and CR values, with the results presented in Table 3. The AVE values for each variable exceed the critical threshold of 0.5, indicating excellent results. Similarly, the CR values exceed the threshold of 0.6, suggesting optimal indicator fit. These findings confirm that the scale demonstrates strong convergent validity.

**Table 3:** Reliability and convergent validity tests.

**Constructs**	**Items**	**Loadings**	**Cronbach's Alpha**	**AVE**	**CR**
PP	PP1	0.757	0.907	0.584	0.908
	PP2	0.752			
	PP3	0.770			
	PP4	0.783			
	PP5	0.766			
	PP6	0.776			
	PP7	0.743			
CA	CA1	0.725	0.811	0.592	0.813
	CA2	0.797			
	CA3	0.784			
WA	WA1	0.751	0.916	0.579	0.917
	WA2	0.750			
	WA3	0.778			
	WA4	0.773			
	WA5	0.785			
	WA6	0.761			
	WA7	0.760			
	WA8	0.725			
PWB	PWB1	0.790	0.871	0.575	0.871
	PWB2	0.738			
	PWB3	0.756			
	PWB4	0.740			
	PWB5	0.765			
WWB	WWB1	0.770	0.886	0.565	0.886
	WWB2	0.740			
	WWB3	0.789			
	WWB4	0.735			
	WWB5	0.728			
	WWB6	0.747			
LGO	LGO1	0.800	0.897	0.636	0.897
	LGO2	0.798			
	LGO3	0.797			
	LGO4	0.802			
	LGO5	0.791			

### 4.3 Common method bias test

Although this study employed data collection methods across multiple time points, common method bias may still exist due to employees' self-assessment of the primary variables. To address this issue, we performed a Harman single-factor analysis using SPSS 27.0 software. The results indicated that the total variance explained was 66.5%, with the first factor accounting for 15.1% of the variance, which is below the recommended threshold of 40% (Podsakoff and Organ, 1986). To further enhance the accuracy of our conclusions, we applied the CMV-ULMC method (controlling for unmeasured latent method factors) using Mplus 8.3 software. We began by adopting the optimal six-factor model from our confirmatory factor analysis as the baseline. Next, we introduced a common method factor (Podsakoff et al., 2003) to create a two-factor model. Finally, we compared the changes in fit indices between the two models to assess model fit. The results revealed that, after adding the common method factor, the model's CFI, TLI, and RMSEA values were 0.989, 0.988, and 0.020, respectively. Compared to the baseline model, the addition of the common method factor did not significantly improve model fit (ΔCFI < 0.1, ΔTLI < 0.1, ΔRMSEA < 0.05, and ΔSRMR < 0.05). Considering all factors, this study did not exhibit significant common method bias, and the data are suitable for further analysis.

### 4.4 Correlation analysis

We conducted a correlation analysis of the primary research variables using SPSS 27.0 software, and the results are presented in Table 4. The findings indicate a significant positive correlation between performance pressure and both challenge appraisal (*r* = 0.340, *p* < 0.01) and workplace anxiety (*r* = 0.379, *p* < 0.01). Similarly, a significant positive correlation exists between challenge appraisal and proactive work behavior (*r* = 0.443, *p* < 0.01), as well as between workplace anxiety and work withdrawal behavior (*r* = 0.405, *p* < 0.01). These results align with our expectations, providing a strong basis for subsequent regression analyses and hypothesis testing.

**Table 4:** Correlation analysis results.

**Variables**	**Mean**	**SD**	**1**	**2**	**3**	**4**	**5**	**6**
1 Performance pressure	3.292	0.918	1					
2 Workplace anxiety	3.320	0.906	0.379^**^	1				
3 Challenge appraisal	3.374	0.977	0.340^**^	0.377^**^	1			
4 Work withdrawal behavior	3.325	0.920	0.351^**^	0.405^**^	0.410^**^	1		
5 Proactive work behavior	3.270	0.973	0.441^**^	0.408^**^	0.443^**^	0.456^**^	1	
6 Learning goal orientation	3.378	1.012	0.204^**^	0.208^**^	0.252^**^	0.235^**^	0.182^**^	1

** p < 0.01.

### 4.5 Hypothesis testing

#### 4.5.1 Testing the mediating influence between challenge evaluation and workplace anxiety

This study first employed the hierarchical regression method to examine the mediating effects. The results regarding the positive coping pathway for performance pressure, presented in Table 5, show that, after controlling for the influence of demographic variables, performance pressure significantly influences challenge appraisal in Model 1 (β = 0.349, *p* < 0.001). Likewise, challenge appraisal positively influences proactive work behavior in Model 6, with a significant effect (β = 0.446, *p* < 0.001). These results support Hypotheses H1 and H2. In Model 5, performance pressure positively affects proactive work behavior (β = 0.457, *p* < 0.001). In Model 7, when both performance pressure and challenge appraisal are included, the mediating variable's effect remains positive and significant (β = 0.331, *p* < 0.001), while the effect of the independent variable is attenuated (β = 0.342, *p* < 0.001). Based on the mediation testing approach proposed by Baron and Kenny (1986), it can be concluded that the mediating variable (challenge appraisal) partially mediates this pathway, thus supporting Hypothesis H3.

**Table 5:** Hierarchical regression analysis results.

**Variable**	**Challenge appraisal**		**Workplace anxiety**			**Proactive work behavior**		**Work withdrawal behavior**		
	**M1**	**M2**	**M3**	**M4**	**M5**	**M6**	**M7**	**M8**	**M9**	**M10**
Gender	0.063	0.061	0.033	0.030	0.043	−0.020	0.022	0.064	0.028	0.054
Age	0.169	0.149	0.215	−0.058	−0.001	−0.094	−0.056	−0.065	−0.162	−0.131
Education	0.023	0.029	0.070	−0.099	−0.021	−0.003	−0.028	0.005	−0.005	−0.017
Enterprise category	−0.073	−0.067	−0.076	0.065	−0.006	0.021	0.018	−0.142^**^	−0.115^*^	−0.118^*^
Position	−0.035	−0.039	−0.096	0.067	−0.149^*^	−0.104	−0.137^*^	−0.086	−0.029	−0.057
Working years	−0.111	−0.097	−0.066	−0.076	0.067	0.148	0.103	0.16	0.207	0.180
Performance pressure	0.349^***^	0.323^***^	0.384^***^	0.345^***^	0.457^***^		0.342^***^	0.354^***^		0.236^***^
Challenge appraisal						0.448^***^	0.331^***^			
Workplace anxiety									0.397^***^	0.308^***^
Learning goal orientation		0.159^**^		0.165^**^						
Performance pressure × Learning goal orientation		0.107^*^		−0.125^*^						
*R* ^2^	0.131	0.174	0.164	0.195	0.207	0.203	0.303	0.152	0.185	0.231
Δ*R*^2^	0.118	0.01	0.142	0.175	0.202	0.198	0.099	0.121	0.154	0.046
*F*	7.462^***^	8.113^***^	9.783^***^	9.340^***^	13.014^***^	12.670^***^	18.819^***^	8.911^***^	11.289^***^	13.041^***^

*** p < 0.001;

** p < 0.01;

* p < 0.05.

The results related to the negative coping pathway for performance pressure, shown in Table 4, demonstrate that, after controlling for demographic variables, performance pressure significantly impacts workplace anxiety in Model 3 (β = 0.384, *p* < 0.001). Moreover, workplace anxiety in Model 9 significantly influences work withdrawal behavior (β = 0.342, *p* < 0.001). These results support Hypotheses H4 and H5. In Model 8, performance pressure positively influences work withdrawal behavior (β = 0.354, *p* < 0.001). In Model 10, when both performance pressure and workplace anxiety are included, the effect of the mediating variable remains positive and significant (β = 0.308, *p* < 0.001), while the effect of the independent variable is weakened (β = 0.236, *p* < 0.001). Applying the mediation testing method proposed by Baron and Kenny (1986), we conclude that the mediating variable (workplace anxiety) partially mediates this path, thus supporting Hypothesis H6.

#### 4.5.2 Test of the moderating influence of learning goal orientation

To examine the moderating effect of learning goal orientation, we performed a hierarchical regression analysis after centering the values of the independent and moderating variables. The results of the analysis are presented in Table 5. Model 2 reveals that learning goal orientation positively moderates the relationship between performance pressure and challenge appraisal, with a significant positive interaction coefficient (β = 0.107, *p* < 0.05). These results support Hypothesis H7. Model 4 indicates that learning goal orientation negatively moderates the relationship between performance pressure and workplace anxiety, with a significant negative interaction coefficient (β = −0.125, *p* < 0.05). These results support Hypothesis H8.

**Figure 2:**
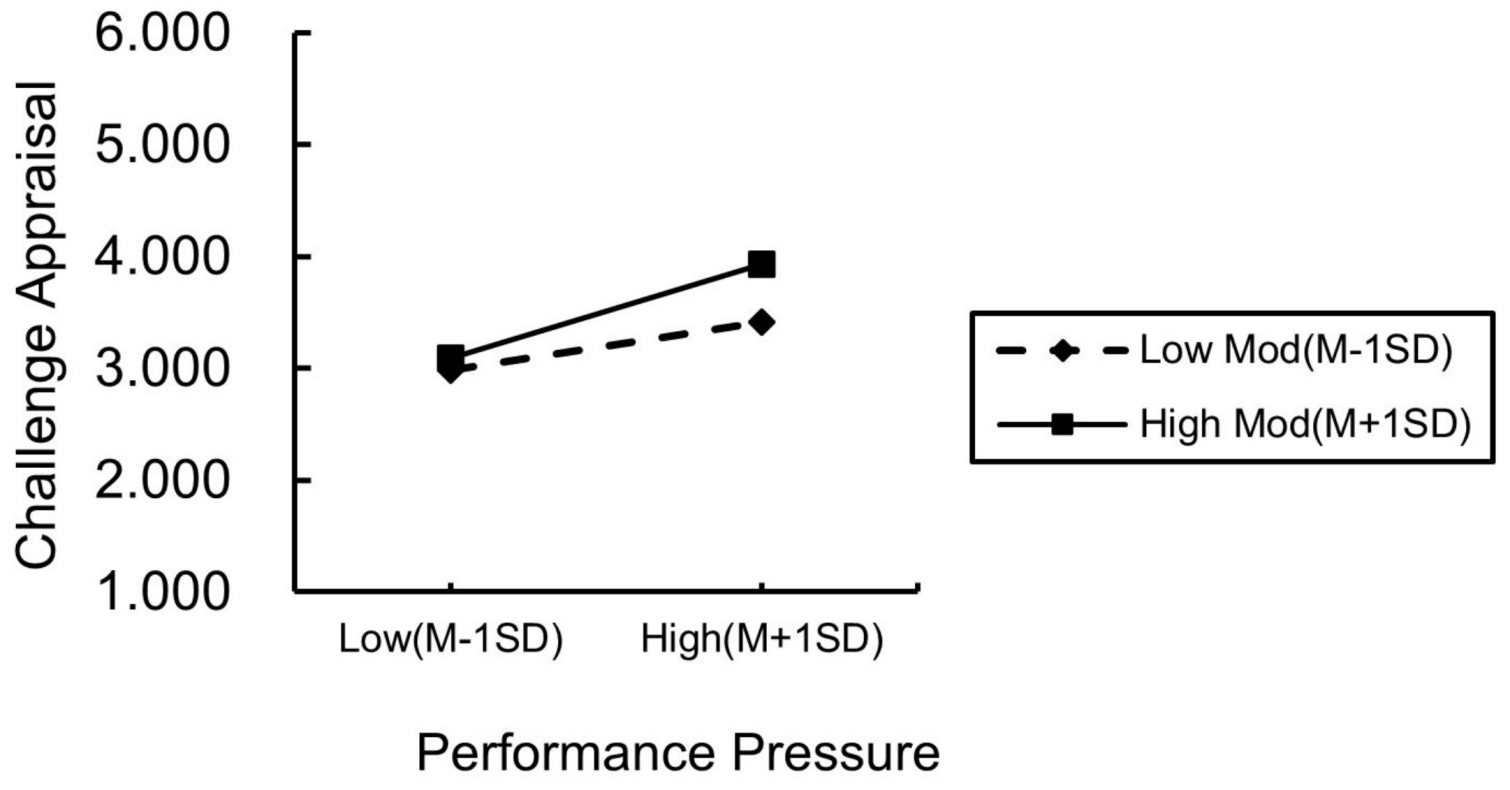
Diagram of the moderating influence of learning goal orientation (CA).

**Figure 3:**
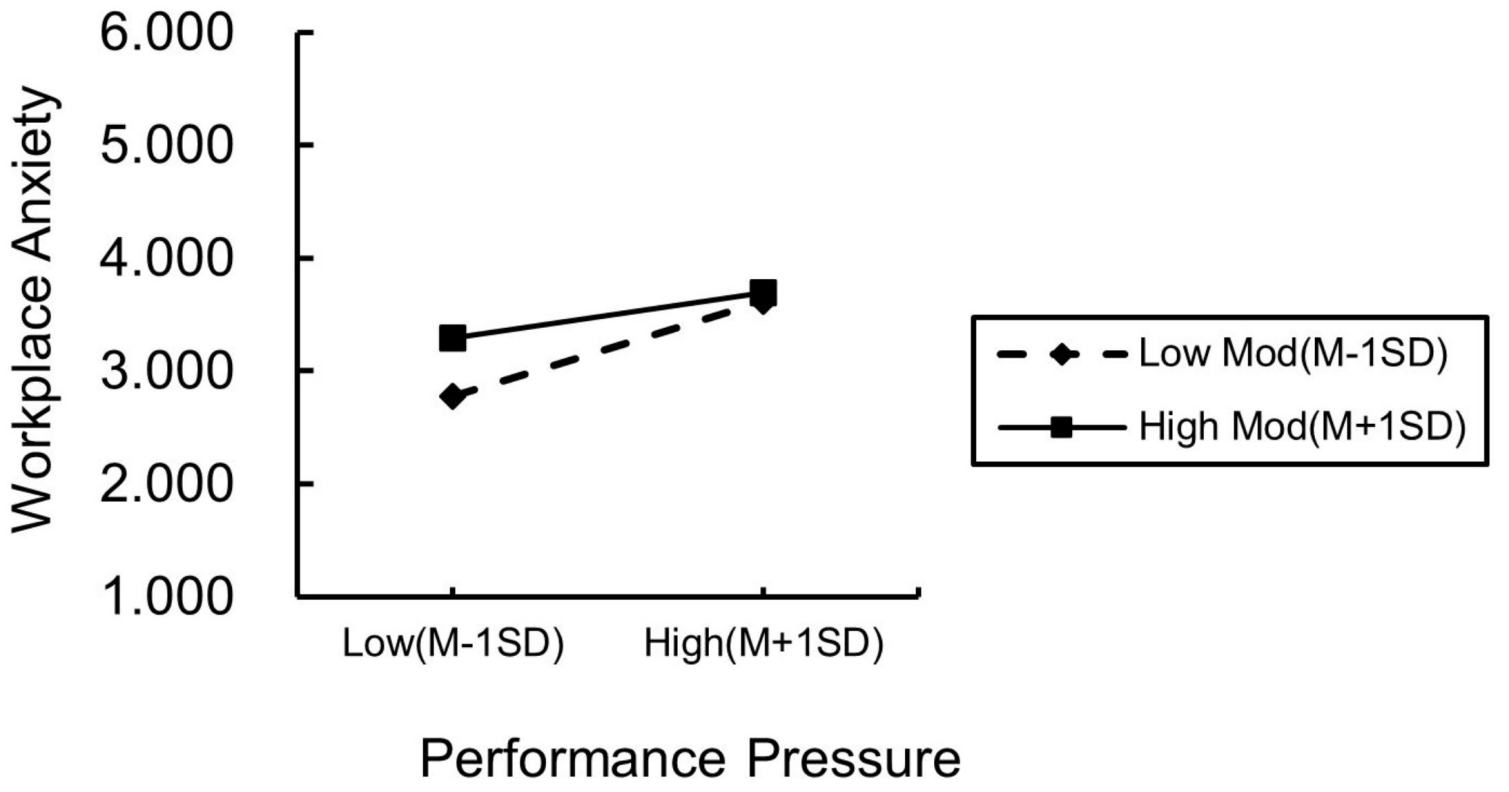
Diagram of the moderating influence of learning goal orientation (WA).

To further explore the moderating role of learning goal orientation, we stratified the levels of learning goal orientation using one standard deviation below the mean as a benchmark. Subsequently, we conducted a simple slope analysis and plotted the interaction effect diagrams. As shown in Figure 2, employees with higher learning goal orientation demonstrate a stronger positive relationship between performance pressure and challenge appraisal compared to those with lower learning goal orientation. Conversely, as shown in Figure 3, employees with higher learning goal orientation exhibit a weaker positive relationship between performance pressure and workplace anxiety compared to those with lower learning goal orientation.

#### 4.5.3 Test of moderated mediation

We continue to apply the Process procedure to perform the bootstrap test for moderated mediation effects. Specifically, the mean of learning goal orientation is adjusted by adding or subtracting one standard deviation, with the results presented in Table 6.

**Table 6:** Results of moderated mediation.

**Path**	**Moderator**	**Effect**	**BootSE**	**BootLLCI**	**BootULCI**
PP → CA → PWB	Lower LGO	0.077	0.029	0.021	0.133
	High LGO	0.150	0.032	0.091	0.217
	Difference	0.073	0.038	0.003	0.152
PP → WA → WWB	Lower LGO	0.144	0.033	0.082	0.212
	High LGO	0.069	0.024	0.025	0.121
	Difference	−0.075	0.033	−0.144	−0.015

N = 356, PP, performance pressure; CA, challenge appraisal; WA, workplace anxiety; PWB, proactive work behavior; WWB, work withdrawal behavior; LGO, learning goal orientation.

Upon examining Table 6, it is evident that when learning goal orientation is low, the estimated effect for the pathway “performance pressure → challenge appraisal → proactive work behavior” is 0.077, with a 95% confidence interval of [0.021, 0.133], which excludes zero. At a high level of learning goal orientation, the effect for the same pathway increases to 0.150, with a 95% confidence interval of [0.091, 0.217], also excluding zero. This indicates a significant mediation effect at high levels of learning goal orientation, and the mediation effect significantly differs between the high and low groups. Therefore, Hypothesis H9 is supported. Furthermore, Table 6 shows that when learning goal orientation is high, the effect for the pathway “performance pressure → workplace anxiety → work withdrawal behavior” is 0.069, with a 95% confidence interval of [0.025, 0.121], which includes zero. However, at a low level of learning goal orientation, the effect for this pathway is 0.144, with a 95% confidence interval of [0.082, 0.212], excluding zero. This suggests a significant negative mediation effect at low levels of learning goal orientation. Notably, the mediation effect differs significantly between the high and low groups. Thus, Hypothesis H10 is also supported.

#### 4.5.4 Path analysis

**Figure 4:**
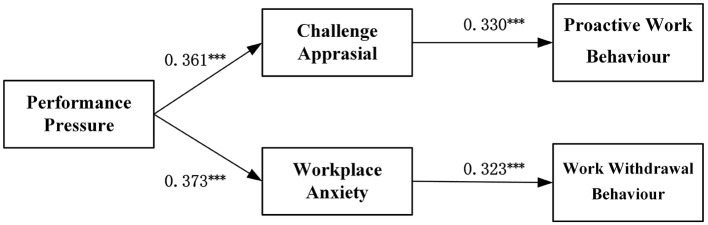
Path analysis coefficient results. ***p < 0.001.

This study employed Mplus 8.3 to perform a comparative test of the mediation effects, aiming to assess the relative strength of two distinct pathways in the model. This approach allows for the simultaneous estimation of multiple regression equations and facilitates the comparison of indirect effects between the two pathways. The results are presented in Figure 4. Specifically, the analysis revealed that the indirect effect of challenge appraisal, linking performance pressure to proactive work behavior, is 0.058. The corresponding 95% confidence interval, ranging from 0.043 to 0.074, excludes zero, indicating a statistically significant indirect effect of challenge appraisal. In contrast, for the pathway between performance pressure and work withdrawal behavior, the indirect effect through workplace anxiety is 0.140, with a 95% confidence interval of [0.110, 0.170], which also excludes zero, confirming the significance of the indirect effect of workplace anxiety. These findings provide further support for hypotheses H3 and H6.

The comparative mediation results are summarized in Table 7. Notably, the difference in the size of the mediation effects between challenge appraisal and workplace anxiety is −0.082, with a confidence interval of [−0.108, −0.056], excluding zero, which indicates a statistically significant difference between the two mediation effects. The mediation effect through the workplace anxiety pathway is more pronounced than that through the challenge appraisal pathway. These results further substantiate hypotheses H3 and H6.

**Table 7:** Results of SEM analyses of multiple mediators.

**Path**	**Estimate**	**95% Confidence interval**	
		**Lower**	**Higher**
CA mediation influence	0.058	0.043	0.074
WA mediation influence	0.140	0.110	0.170
Contrasting mediating influences (CA-WA)	−0.082	−0.108	−0.056

CA, challenge appraisal; WA, workplace anxiety.

## 5 Discussion

Building upon the transactional theory of stress and the affective event theory, our study demonstrates that performance pressure indirectly promotes proactive work behavior through challenge appraisal. Conversely, it also indirectly leads to work withdrawal behavior due to the influence of workplace anxiety. These findings elucidate the differential responses to performance pressure and the mechanisms underlying these behaviors. In practice, our results show that workplace anxiety exerts a stronger impact than challenge appraisal, indicating that emotions play a crucial role in shaping individuals' reactions to stress. Moreover, possessing a learning goal orientation fosters more positive appraisals of performance pressure and mitigates its negative emotional effects. This discovery expands our understanding of the factors that can either exacerbate or alleviate stress. Overall, these findings offer valuable theoretical and practical insights into the ways in which performance pressure influences employee behavior, emphasizing the pivotal roles of cognition and emotion in effective coping strategies.

### 5.1 Theoretical contributions

This study makes three significant theoretical contributions. First, it reveals the dual nature of performance pressure in shaping employees' work behavior. Prior research has identified both positive and negative effects of performance pressure. For instance, Tang et al. (2023) highlighted its potential to induce negative coping and unethical pro-organizational behavior, while He et al. (2024) demonstrated that it can stimulate positive coping and foster growth at work. Our study shows that these divergent influences can occur simultaneously. By considering the dual nature of performance pressure, we integrate findings from previous studies that largely examined this phenomenon from a singular perspective. Recognizing that performance pressure can both motivate and demotivate employees, we propose a dual approach that transcends traditional models. Furthermore, our research extends the application of the transactional theory of stress and the affective event theory to a new context. By combining these theoretical frameworks, we provide a deeper understanding of how individuals cognitively appraise performance pressure and the emotional reactions and behaviors that stem from these appraisals. The dual-path approach we propose not only captures the complexity of performance pressure's impact on employees but also offers a more comprehensive understanding of the underlying mechanisms. This approach highlights that performance pressure can either stimulate adaptive coping strategies and positive outcomes, such as increased effort and improved job performance, or trigger maladaptive responses and negative consequences, such as burnout and reduced job satisfaction. By distinguishing these dual pathways, we provide a new perspective that can help resolve inconsistencies in prior research, which often reported mixed findings due to a failure to consider performance pressure's dual nature. Additionally, our work lays the groundwork for future research to explore interventions and strategies that can leverage the motivating potential of performance pressure while mitigating its detrimental effects, ultimately fostering a healthier and more productive workplace environment.

Second, drawing on the transactional theory of stress and the affective event theory, this study examines how performance pressure influences employees' work behavior through both cognitive and emotional pathways. We adopt a comprehensive perspective to explore how employees react under such conditions by considering their cognitive appraisals and emotional responses to performance pressure. Recent research has emphasized the cognitive effects of performance pressure on employee behavior. For instance, Kundi et al. (2021) found that performance pressure influences work engagement through challenge and threat appraisals, while Kumar and Lavanya (2024) demonstrated that it fosters employee innovation through feedback-seeking behavior. However, less attention has been given to the emotional pathways, particularly how performance pressure induces workplace anxiety, which can lead to work withdrawal. Building on previous studies and the rationale of this research, we propose that performance pressure has a “double-edged sword” effect on employee behavior. This novel approach provides a more detailed and comprehensive understanding of how employees cope with performance pressure through both cognitive restructuring and emotional regulation. Our findings contribute to the multidimensional discourse on the mechanisms of performance pressure and offer a new perspective for the academic community in understanding its impact on employee behavior.

Third, this study investigates the moderating effect of learning goal orientation and identifies the boundary conditions through which performance pressure influences employees' cognition and emotions. This contributes a novel perspective to the study of performance pressure. Existing research has primarily focused on factors such as leadership style and organizational support as boundary conditions for the impact of performance pressure (Wang et al., 2021). However, there has been limited exploration of how individual traits influence these dynamics. For example, Kundi et al. (2021) found that emotional stability shapes individuals' cognitive appraisals of performance pressure. Building on their work, this study introduces learning goal orientation into the workplace context and examines its moderating role. This provides fresh insights into managing performance pressure. Employees with varying levels of learning goal orientation demonstrate differences in their cognitive and emotional responses to performance pressure. Specifically, this study finds that employees with a high learning goal orientation are more likely to perceive the potential benefits of performance pressure and engage in challenge appraisals. In contrast, employees with a low learning goal orientation tend to focus on the threats posed by performance pressure and experience workplace anxiety. By examining individual differences, this study clearly defines the boundary conditions of performance pressure's influence on employee behavior outcomes. This enriches the theoretical framework surrounding the role of learning goal orientation in the workplace.

### 5.2 Practical implications

The implications for organizational management practice can be summarized in three key points. First, this study provides valuable insights into the management of organizational performance pressure. Both organizations and employees must acknowledge performance pressure as a pervasive source of stress within contemporary workplaces. A more effective response to performance pressure can be achieved by recognizing its dual impact. As employees vary in their cognitive appraisals, they respond differently to performance pressure; therefore, organizational management must adopt personalized strategies tailored to individual needs. For example, leaders can consider the unique differences among employees when guiding them in managing performance pressure. On one hand, the challenges and positive aspects of performance pressure can motivate employees and unlock their potential. On the other hand, coping with performance pressure depletes cognitive and emotional resources. Extended resource depletion cannot be easily replenished, leading to inevitable negative consequences. Organizations must understand the intricate relationship between performance pressure and employees' active behaviors, allowing them to effectively harness performance pressure to maintain employee motivation and performance. For employees, accurately assessing their abilities and making appropriate cognitive appraisals are crucial for managing performance pressure, which can prevent avoidance behaviors that lead to physical and mental exhaustion.

Second, organizational managers must recognize the critical importance of regulating the psychological wellbeing of their employees. While workplace anxiety can motivate employees to enhance their skills, excessive anxiety can lead to decreased work performance and an increase in unethical behavior. Therefore, it is essential for managers to prioritize understanding and supporting employees' psychological wellbeing and emotional health. Establishing effective communication channels and a robust psychological support system is crucial. This approach can help alleviate workplace anxiety promptly, ensuring it remains within a manageable range, thereby enabling employees to better cope with work-related stress and challenges. By fostering a supportive environment that prioritizes mental health, organizations can create a healthier and more harmonious workplace. This, in turn, can enhance employee job satisfaction and productivity, contributing to the long-term sustainable development of the organization.

Third, organizations can enhance employee motivation by fostering a strong learning goal orientation. The findings suggest that employees with a high learning goal orientation are more inclined to engage in challenge appraisals. When facing performance pressure, these individuals tend to respond proactively to sudden threats, as they are less susceptible to negative environmental influences. Managers can pay particular attention to the psychological states of employees with low learning goal orientation and implement targeted training and guidance measures. For instance, employees can enhance their ability to withstand pressure and adapt to challenging environments by engaging in activities such as competitive sports. Since employees with high learning goal orientation are more likely to actively confront performance pressure, leaders can actively encourage and support them in achieving their goals, thereby promoting the optimal allocation of tasks. Previous research has shown that developmental leadership, and beliefs about effort and ability are positively correlated with learning goal orientation (Vandewalle et al., 2019). Consequently, organizations can enhance leadership practices and foster a culture of employee development through targeted leadership training. During recruitment, companies can prioritize candidates with a strong willingness to learn. Furthermore, providing diverse knowledge and skills training programs can create conducive learning environments, helping employees establish appropriate learning goal orientations. Simultaneously, organizations can strive to cultivate a high-level learning atmosphere to support continuous growth and development.

### 5.3 Limitations and future research

First, while this study employs two-wave data collection, it remains a cross-sectional study by design. Future research should replicate these findings using alternative methodologies, such as experimental or longitudinal designs, to strengthen causal inferences and address potential common method bias. This study ultimately collected 356 valid responses, but the sample was limited to employees in China. Consequently, it does not account for the variations in population demographics, work environments, and cultural contexts across different countries and regions. Future studies should consider these differences to broaden the generalizability and representativeness of the findings.

Second, based on the dual-path theory integrating cognition and emotion, this study explored the mediating roles of challenge appraisal and workplace anxiety in the relationship between performance pressure and employee behavioral outcomes. However, this study does not exhaustively examine other potential mechanisms through which performance pressure may affect employees. Future research could explore additional mediating mechanisms from diverse theoretical perspectives, such as negative rumination, emotional exhaustion, and other possible mediating variables. This could further enrich the understanding of how performance pressure influences employee behavior.

Third, this study introduces learning goal orientation as a moderating variable from the perspective of individual personality traits. Future studies could expand on this by investigating additional moderating variables based on organizational environmental factors. For example, the achievement context could play a critical role in shaping the impact of performance pressure. In achievement-oriented environments, individuals may be more focused on performance outcomes, thereby increasing performance pressure. In contrast, more inclusive and diverse work environments may encourage a focus on intrinsic motivations and personal interests, potentially reducing the emphasis on performance (Tang et al., 2023). Incorporating environmental factors, such as motivational climate, as moderators could provide further insights into the boundary conditions of performance pressure (Vandewalle et al., 2019). Therefore, future research should explore a broader range of organizational environmental factors to gain a more nuanced understanding of how performance pressure influences employee behavior. This would offer valuable practical implications for organizational management, as these factors may either amplify or mitigate the effects of performance pressure.

## 6 Conclusion

In summary, this study develops a moderated dual-mediation model based on the transactional theory of stress and the affective event theory, offering valuable insights into the complex behavioral responses of employees facing performance pressure. Our findings reveal that proactive work behavior, which reflects the relentless drive and competition akin to the “Rat Race,” contrasts with work withdrawal behavior, characterized by resignation and passivity, as seen in the “Lying Flat” phenomenon. The results clearly demonstrate that the impact of performance pressure on employees' work behavior is contingent on their individual cognitive appraisals, particularly their challenge appraisals, as well as their emotional responses, particularly workplace anxiety. Additionally, this study highlights the moderating role of learning goal orientation in shaping both employees' cognitive appraisals and their emotional responses to performance pressure. Consequently, whether employees engage in the persistent “Rat Race” or adopt the passive “Lying Flat” approach depends on these cognitive and emotional factors. Our research provides a novel perspective for organizations seeking to effectively balance and manage employees' performance pressure, helping to cultivate an environment that promotes both productivity and wellbeing.

## Data Availability

The original contributions presented in the study are included in the article/supplementary material, further inquiries can be directed to the corresponding author.
